# Traditional Chinese Medicine for Acute Myocardial Infarction in Western Medicine Hospitals in China

**DOI:** 10.1161/CIRCOUTCOMES.117.004190

**Published:** 2018-03-16

**Authors:** Erica S. Spatz, Yongfei Wang, Adam L. Beckman, Xuekun Wu, Yuan Lu, Xue Du, Jing Li, Xiao Xu, Patricia M. Davidson, Frederick A. Masoudi, John A. Spertus, Harlan M. Krumholz, Lixin Jiang

**Affiliations:** From the Center for Outcomes Research and Evaluation, Yale-New Haven Hospital, CT (E.S.S., Y.W., Y.L., X.X., H.M.K.); Section of Cardiovascular Medicine, Department of Internal Medicine (E.S.S., Y.W., H.M.K.), Department of Obstetrics and Gynecology and Reproductive Sciences (X.X.), Yale School of Medicine, New Haven, CT; Harvard Medical School, Boston, MA (A.L.B.); National Center for Cardiovascular Diseases, Fuwai Hospital, Beijing, China (X.W., X.D., J.L., L.J.); Johns Hopkins School of Nursing, Baltimore, MD (P.M.D.); University of Colorado Anschutz Medical Campus and the Colorado Cardiovascular Outcomes Research Consortium, Denver (F.A.M.); Saint Luke’s Mid America Heart Institute/University of Missouri – Kansas City (J.A.S.); and Department of Health Policy and Administration, Yale School of Public Health, New Haven, CT (H.M.K.).

**Keywords:** cardiovascular diseases, complementary therapies, medicine, Chinese traditional, quality of health care, Salvia miltiorrhiza

## Abstract

**Background:**

Amid national efforts to improve the quality of care for people with cardiovascular disease in China, the use of traditional Chinese medicine (TCM) is increasing, yet little is known about its use in the early management of acute myocardial infarction (AMI).

**Methods and Results:**

We aimed to examine intravenous use of TCM within the first 24 hours of hospitalization (early IV TCM) for AMI. Data come from the China Patient-centered Evaluative Assessment of Cardiac Events Retrospective Study of Acute Myocardial Infarction, restricted to a large, representative sample of Western medicine hospitals throughout China (n=162). We conducted a chart review of randomly sampled patients with AMI in 2001, 2006, and 2011, comparing early intravenous TCM use across years, predictors of any early intravenous TCM use, and association with in-hospital bleeding and mortality. From 2001 to 2011, early intravenous TCM use increased (2001: 38.2% versus 2006: 49.1% versus 2011: 56.1%; *P*<0.01). Nearly all (99%) hospitals used early intravenous TCM. Salvia miltiorrhiza was most commonly prescribed, used in one third (35.5%) of all patients admitted with AMI. Patients receiving any early intravenous TCM, compared with those who did not, were similar in age and sex and had fewer cardiovascular risk factors. In multivariable hierarchical models, admission to a secondary (versus tertiary) hospital was most strongly associated with early intravenous TCM use (odds ratio: 2.85; 95% confidence interval: 1.98–4.11). Hospital-level factors accounted for 55% of the variance (adjusted median odds ratio: 2.84). In exploratory analyses, there were no significant associations between early intravenous TCM and in-hospital bleeding or mortality.

**Conclusions:**

Early intravenous TCM use for AMI in China is increasing despite the lack of evidence of their benefit or harm. There is an urgent need to define the effects of these medications because they have become a staple of treatment in the world’s most populous country.

**Clinical Trial Registration:**

URL: https://www.clinicaltrials.gov. Unique identifier: NCT01624883

## Abstract 2

Supplemental Digital Content is available in the text.

**WHAT IS KNOWN**

- The use of traditional Chinese medicine to prevent and treat a broad range of conditions, including cardiovascular disease, is rapidly growing in China.
- Traditional Chinese medicine is being used in Western medicine hospitals for the treatment of acute myocardial infarction although limited data are available about the types of traditional Chinese medicines used and whether they are used alone or as a complementary therapy.
- Chinese guidelines for the treatment of acute myocardial infarction are closely aligned with the US and European guidelines and do not discuss the use of traditional Chinese medicine.

**WHAT THE STUDY ADDS**

- In a large, nationally representative study of acute myocardial infarction care in Western medicine hospitals throughout China, use of traditional Chinese medicine for acute myocardial infarction increased from 2001 to 2011, with over half of patients treated with intravenous formulations of traditional Chinese medicine in the first 24 hours of hospitalization.
- Nearly all hospitals in the study used traditional Chinese medicine in at least some patients although secondary care hospitals (versus tertiary care hospitals) were more likely to use traditional Chinese medicine. Overall, hospital-level factors accounted for 55% of the variance.
- Traditional Chinese medicine was used in conjunction with guideline-based medical therapies and not as a stand-alone treatment.
- In this study, we did not find an association with in-hospital bleeding or mortality, although more research is needed.

Traditional Chinese medicine (TCM), a treatment approach that evolved over centuries to prevent and treat disease, incorporates herbal medicines and mind-body practices and remains an integral part of the Chinese healthcare system. Even as the healthcare market for Western therapies has expanded, so has the market for TCM.^1^ In 2011, $64 billion dollars was spent on TCM, an increase of ≈20% from 2008, with the greatest demand for TCM in the treatment of cardiac and cerebrovascular diseases.^2^ Despite the substantial increase in use, little is known about practice patterns for treating acute cardiovascular disease with TCM.

In China, the treatment of acute myocardial infarction (AMI) is mainly delivered in modern Western medicine hospitals that emphasize conventional therapies (as opposed to traditional or integrative Chinese hospitals that emphasize TCM); nonetheless, use of TCM among Western medicine hospitals is common.^3^ TCMs are reported to impact platelet aggregation, circulation, oxidative stress, vasoreactivity, and other cardiovascular properties,^4,5^ yet studies of the effectiveness of these agents are methodologically limited,^4,6^ and systematic reviews of TCM for cardiovascular disease have been inconclusive.^5,7,8^ In addition, although TCM has been used for centuries, there is no mention of TCM in the Chinese guidelines for treating AMI,^9–11^ which closely align with those from the United States and Europe. Thus, although TCMs are used in the care of patients with AMI, there is limited data about their use, with implications for disease management and outcomes. Specifically, we lack data on the types of TCM used to treat AMI and whether they are being used alone or in conjunction with conventional treatments.^10,12–16^

Accordingly, we used a nationally representative sample of patients with AMI admitted to Western medicine hospitals throughout China to investigate trends and outcomes associated with TCM, with a focus on intravenous therapies used in the first 24 hours (early intravenous TCM) to ensure that it was the intention of the providers to use TCM as opposed to patients supplementing their care with their own treatments. In addition, market data show that spending on intravenous TCM exceeds that of oral TCM for cardiovascular disease management.^2^ Our primary aims were to (1) describe practice patterns in the use of early intravenous TCM across 3 study periods: 2001, 2006, and 2011; and (2) assess patient and hospital-level characteristics associated with early intravenous TCM use. In a secondary exploratory analysis, we assessed signals for harm and benefit by examining the association of early intravenous TCM with in-patient bleeding and mortality. Findings from this study are important for supporting quality improvement efforts in China that are sensitive to the role of TCM as part of China’s history and culture and yet simultaneously commit to the safe and effective use of evidence-based approaches to improve outcomes for people with AMI.

## Methods

It is our goal to share the China PEACE-Retrospective AMI Study (China Patient-centered Evaluative Assessment of Cardiac Events Retrospective Study of Acute Myocardial Infarction) data; however, at this time, we are unable to do so.

### Study Design, Setting, and Cohort

**Figure 1.**
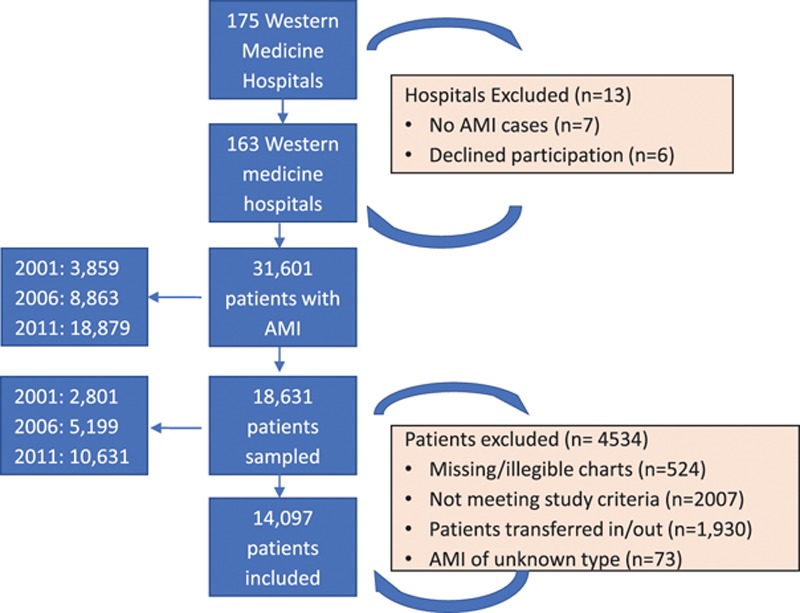
**The China PEACE (Patient-centered Evaluative Assessment of Cardiac Events) retrospective study of acute myocardial infarction: study flow chart**. AMI indicates acute myocardial infarction.

The China PEACE-Retrospective AMI Study was designed to assess trends in quality and outcomes of patients hospitalized with AMI during 3 different time periods in China: 2001, 2006, and 2011.^17^ The study used a 2-stage approach to sampling described in detail by Dharmarajan et al.^17^ Briefly, we first identified a nationally representative cohort of hospitals from 5 regions, selected based on the 3 official economic-geographic regions of Mainland China (Eastern-rural, Central-rural, Western-rural, Eastern-urban, and Central/Western-urban). In total, 175 modern Western medicine hospitals (a phrase used in China to designate hospitals that are based in Western or conventional therapies, distinguishing them from traditional and integrative hospitals) were sampled (105 rural and 70 urban); 7 had no admissions for AMI and 6 declined to participate, resulting in a final cohort of 162 hospitals. In the second stage, systematic random sampling was used to identify hospitalizations with a principal diagnosis of AMI, defined through *International Classification of Diseases*—Clinical Modification codes versions 9 (410.xx) and 10 (I21.xx), or principal discharge diagnosis terms noted in the medical record. From a total of 31 601 patients with AMI across the 3 study years, 18 631 patients were sampled using systematic random sampling procedures. After excluding patients with missing or illegible medical charts (n=524), patients not meeting study criteria (n=2007), patients transferred in or out (n=1930) of the current hospital, and AMIs of unknown type (ie, neither ST-segment–elevation myocardial infarction nor non–ST-segment–elevation myocardial infarction; n=73), the final cohort included 14 097 patients (Figure 1).

Medical charts were reviewed by a centralized data abstraction team using standardized data definitions. Rigorous quality standards were used, and 5% of medical records were randomly audited, with overall variable accuracy of >98%.^17^ Hospital characteristics were collected via an electronic questionnaire completed by investigators at each participating hospital.

### Classification of TCM

Herbal medicines, including the name, route of administration, and timing of use in the course of hospitalization, were abstracted from the medical record. Use of nonpharmacological TCM (eg, qigong; acupuncture) for AMI is limited, if at all used, and therefore was not abstracted. Herbal medicines were classified into 6 main groups based on the primary active ingredient (Table I in the Data Supplement). The route of administration was categorized as either oral or intravenous, and timing of administration was categorized as either within first 24 hours or later. We focused on the most commonly used early intravenous TCMs (defined as being used in >5% of patients): Salvia miltiorrhiza; Folium ginkgo; and Panax notoginseng. TCMs that were infrequently used were combined into a category, labeled: Other TCM.

### Covariates

We assessed the following variables, hypothesized to be associated with TCM use: year of AMI hospitalization; demographics (age and sex); clinical characteristics (presenting signs and symptoms; baseline cardiovascular risk factors and disease; AMI type and severity; and eligibility for reperfusion); clinical care management (pharmacological and reperfusion therapies—including receipt and timing of percutaneous coronary intervention [PCI] or fibrinolysis); and hospital-level characteristics (economic–geographic region; acuity status, ie, tertiary versus secondary; capacity to perform PCI; presence of coronary care unit). For variables with <1% missing, we imputed the values based on the most common values for categorical variables and the median value for continuous variables. For variables missing >1%, we created a dummy variable indicating missing and included it with the original variable in the adjusted models.

### Outcomes

Patient outcomes were assessed in a composite measure of in-hospital mortality or withdrawal from treatment. Withdrawal from treatment is a common disposition among patients deemed to be actively dying, but who do not want to die in the hospital; this disposition status was adjudicated by cardiologists in the coordinating study center. The outcome of in-hospital mortality or treatment withdrawal is used as a quality measure for hospitals by the Chinese government.^18^ We also assessed in-hospital bleeding given the antiplatelet effects of many of the TCMs and the potential for bleeding, especially if combined with other antiplatelet or anticoagulant use. Major bleeding was defined as any intracranial bleeding, an absolute decrease in hemoglobin of 5 mg/dL, bleeding resulting in hypovolemic shock, or fatal bleeding resulting in death within 7 days.

### Statistical Analysis

We describe the frequency of early intravenous TCM use among patients admitted with AMI across the 3 study periods, including TCM type, timing of administration (proportion administered in the first 24 hours of hospitalization), and potential cardiac effects for the most common TCMs used. Trends in early intravenous TCM were assessed with the Mann–Kendall test for continuous variables. We examined pairwise correlations to assess commonly used combinations of the TCMs used in the first 24 hours; specifically, we calculated the frequency of use of each pair of the 4 TCMs and calculated the Pearson correlation coefficient to examine the association between each TCM pair. In addition, we compared patient and hospital-level characteristics by the use of early intravenous TCM.

To identify factors associated with early intravenous TCM, we used hierarchical logistic regression models with backward stepwise selection of covariates, using a random effect at the hospital level to account for the patients’ clustering within hospitals. To assess whether early intravenous TCM was used in lieu of evidence-based therapies, we assessed the bivariate and multivariable association of early intravenous TCM with evidence-based, pharmacological therapies (aspirin, β-blocker, statin, heparin, clopidogrel, angiotensin-converting enzyme inhibitor/angiotensin receptor blocker), use of biomarker testing, cardiac catheterization, echocardiography, and reperfusion (PCI and fibrinolysis). To estimate the variability in hospital-level early intravenous TCM use, we calculated the median odds ratio (OR) from the fully adjusted hierarchical model, which represents the average likelihood of a statistically identical patient receiving early intravenous TCM at one random hospital versus another.

In exploratory analyses, we compared outcomes of patients receiving any early intravenous TCM, along with specific types of TCM using hierarchical logistic regression models adjusted for patients’ demographic and admission characteristics, medical history, diagnostic procedures, in-hospital medications and procedures, and hospital characteristics. This study was approved by the institutional review boards of Yale University and the Fuwai Hospital, National Center for Cardiovascular Disease in China.

## Results

### Utilization Patterns of Early Intravenous TCM

Among the 14 097 patients admitted with AMI across the 3 time periods (representing 223 286 patients with AMI in 2011), two thirds (n=9424; 66.9%) received either oral or intravenous TCM during hospitalization, among which 77% was administered intravenously within the first 24 hours of hospitalization (Table 1; Table II in the Data Supplement). Use of early intravenous TCM increased significantly across the 3 time periods: 38.2% in 2001 versus 49.1% in 2006 versus 56.1% in 2011; *P*<0.001. Among hospitals enrolled in 2011 (n=162), all but 2 hospitals administered early intravenous TCM.

**Table 1.**
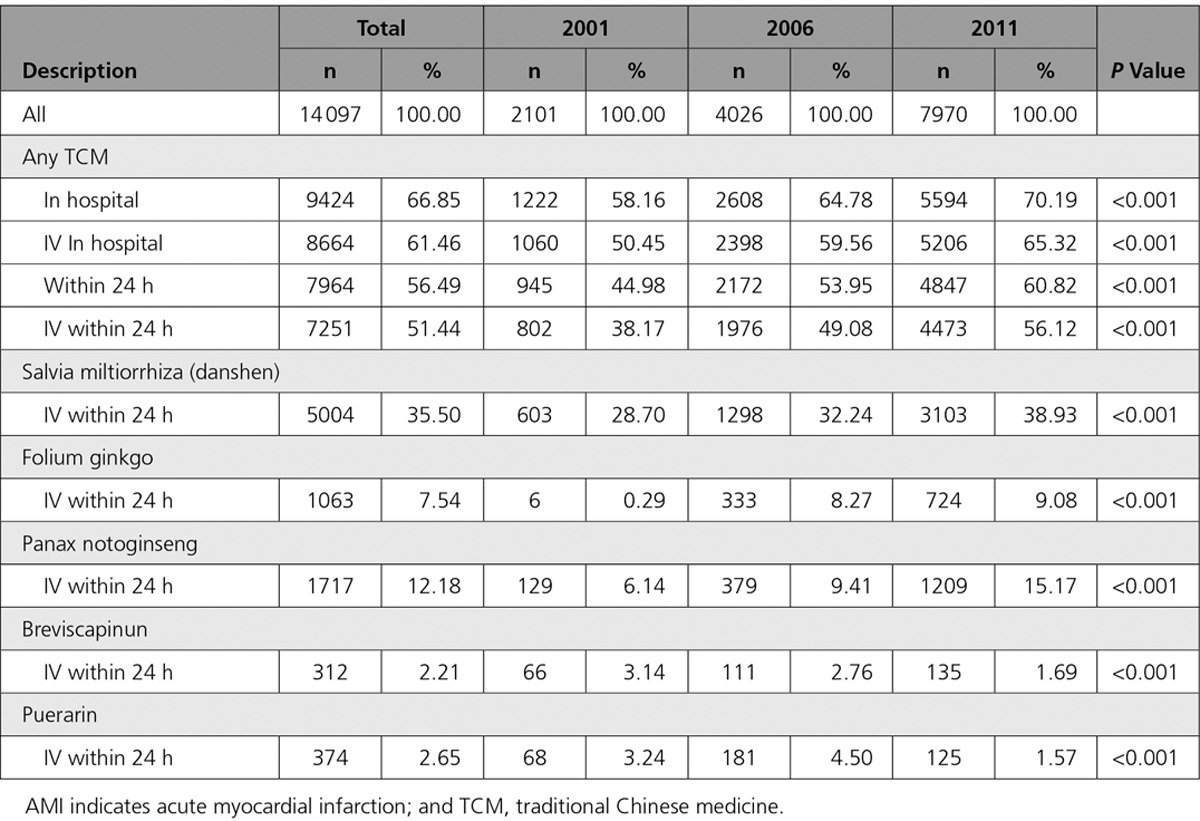
Trends in Early intravenous TCM Use Among Patients Treated for AMI in Western Medicine Hospitals in China

The most commonly used early intravenous TCMs were Salvia miltiorrhiza (danshen), used in one third (35.5%) of all patients admitted with AMI, followed by Panax notoginseng (12.2%); Folium ginkgo (7.5%); puerarin (2.7%); and breviscapinun (2.2%). Nine percent of patients received ≥2 types of early intravenous TCM, with the most common combinations being Salvia miltiorrhiza (danshen) with either Panax notoginseng (4.0%) or Folium gingko (2.5%).

### Characteristics of Patients Receiving Early Intravenous TCM

Characteristics of patients who did and did not receive early intravenous TCM are compared in Table 2. The mean age of patients was similar (65.3 years [SD: 12.4] versus 65.2 years [SD: 12.6]), and there were no sex differences (% female: 30.9% versus 32.0%). Statistically significant, but clinically modest differences in baseline characteristics were observed. Patients receiving any early intravenous TCM were less likely to have a prior diagnosis of angina or coronary heart disease (21.9% versus 23.8%), prior AMI (9.8% versus 11.8%), or to have hypertension, diabetes mellitus, hyperlipidemia, or be active smokers at baseline. On presentation, patients receiving early intravenous TCM were more likely to present with ST-segment–elevation myocardial infarction (87.1% versus 84.6%) and within 3 hours of symptom onset (20.6% versus 17.0%); they were less likely to present after 72 hours of symptom onset (26.5% versus 31.8%). Mean presenting systolic blood pressure was lower among people receiving early intravenous TCM (126 versus 130 mm Hg). Patients receiving early intravenous TCM were more likely to present in cardiogenic shock (6.6% versus 4.9%).

**Table 2.**
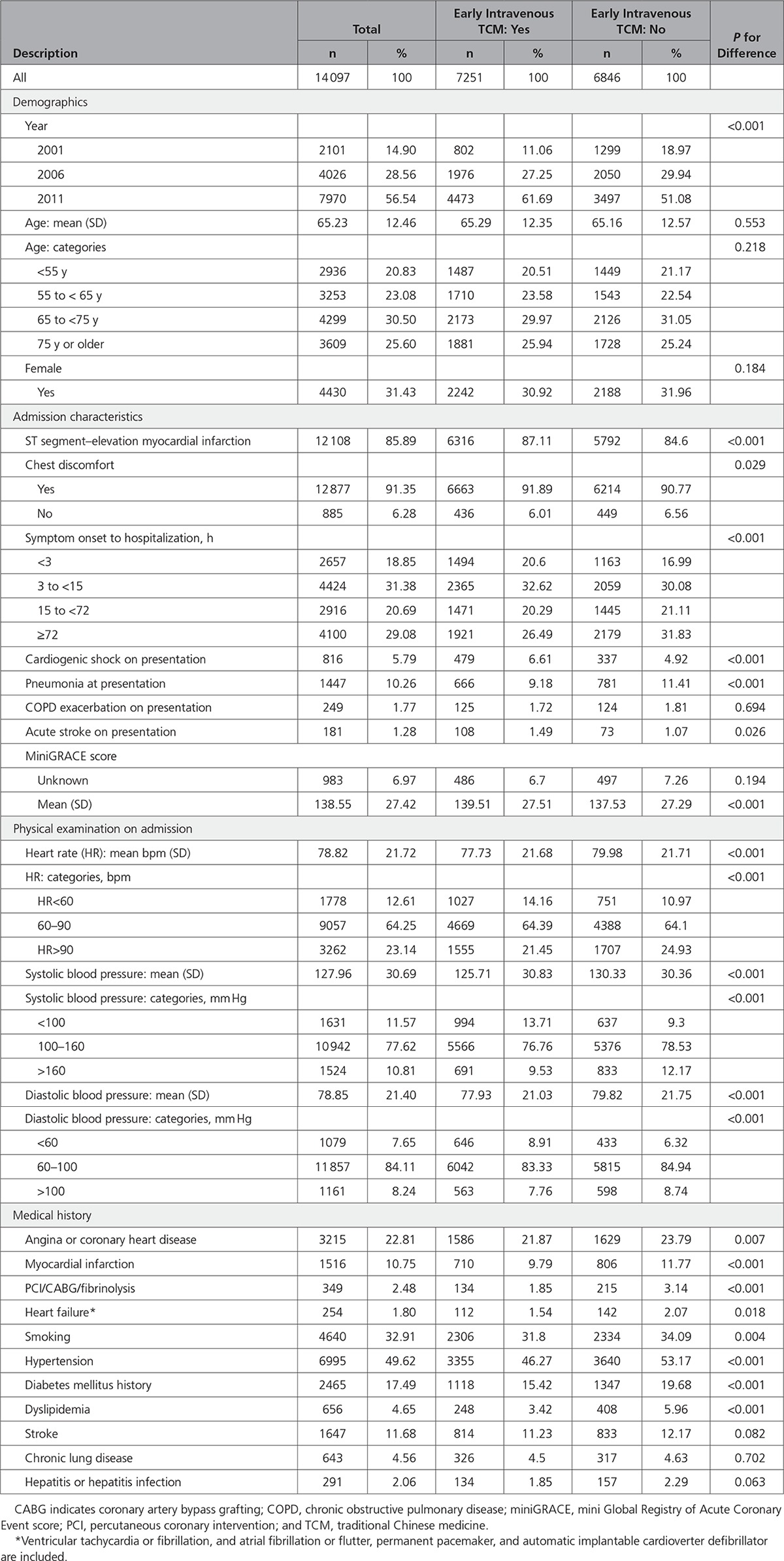
Baseline Characteristics Stratified by Use of Intravenous TCM Within 24 Hours

Bivariate analyses revealed that in-hospital management differed between patients receiving early intravenous TCM and patients who did not (Table 3). Although there were no differences in reperfusion rates (30.2% versus 30.6%), there were significant unadjusted differences in the types of reperfusion among the 2 groups, with fewer primary PCI (6.3% versus 15.0%) and more fibrinolysis therapy (23.9% versus 15.6%) in the early intravenous TCM group. Comparing early intravenous TCM with no early intravenous TCM, more patients received aspirin (88.4% versus 82.9%), statin (71.3% versus 63.2%), and heparin (80.3% versus 69.9%), with no difference in angiotensin-converting enzyme inhibitor/angiotensin receptor blocker (47.8% versus 48.5%), clopidogrel (56.4% versus 54.8%), or β-blocker (46.3% versus 47.2%).

**Table 3.**
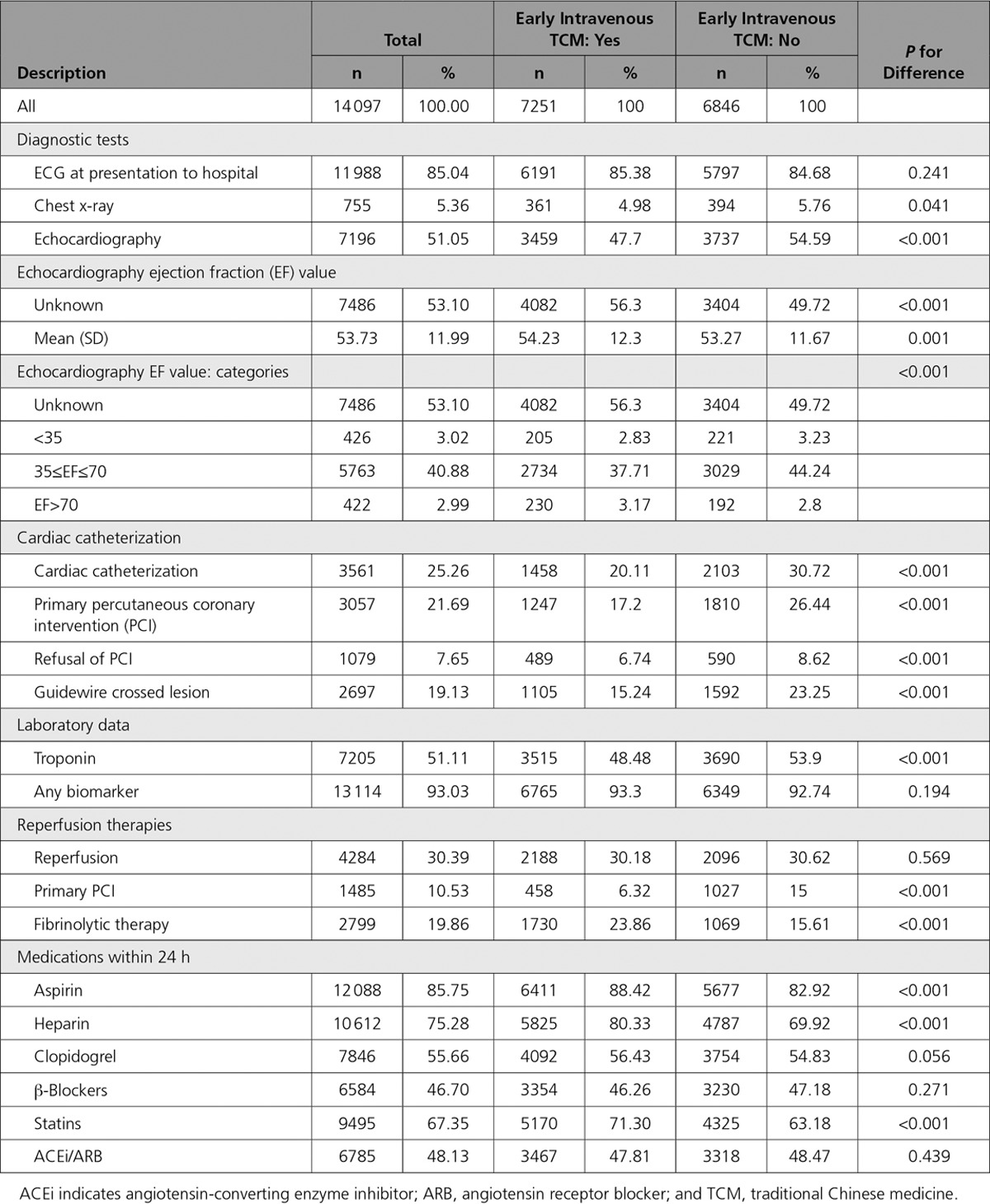
Clinical Management Stratified by Use of Intravenous TCM Within 24 Hours

### Patient and Hospital Characteristics Associated With Early Intravenous TCM Administration

**Figure 2.**
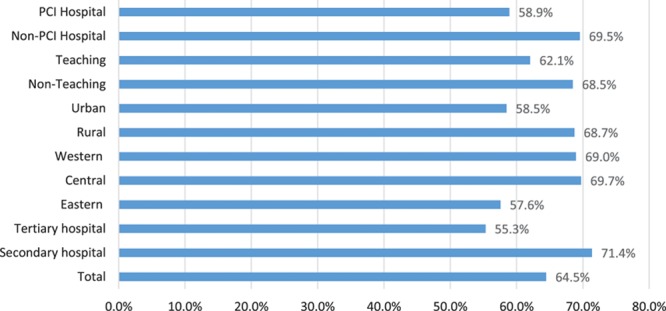
**Hospital characteristics associated with use of early intravenous traditional Chinese medicine in patients with acute myocardial infarction in 2011.**

Although nearly all of the sampled hospitals used early intravenous TCM in at least some patients with AMI, there were significant differences in the magnitude of patients receiving early intravenous TCM among different hospitals (mean, 63.0%; SD, 24.1%; Table 4). Certain hospital characteristics were associated with greater use of early intravenous TCM (Figure 2). Secondary hospitals administered more early intravenous TCM than tertiary hospitals (mean use in 2011: 71.4%; SD 22.8% versus 55.3%; SD, 27.7%). Early intravenous TCM was also used more commonly in nonteaching (68.4%; SD, 23.8%) versus teaching hospitals (62.1%; SD, 27.4%), non-PCI (69.5%; SD, 22.1%) versus PCI hospitals (58.9%; SD, 29.2%), rural (68.7%; SD, 24.4%) versus urban hospitals (58.5%; SD, 27.6%), and among hospitals in the Central (69.7%; SD, 19.8%) and Western (69.0%; 25.8%) regions, as compared with the Eastern region (57.6%; SD, 29.3%).

**Table 4.**
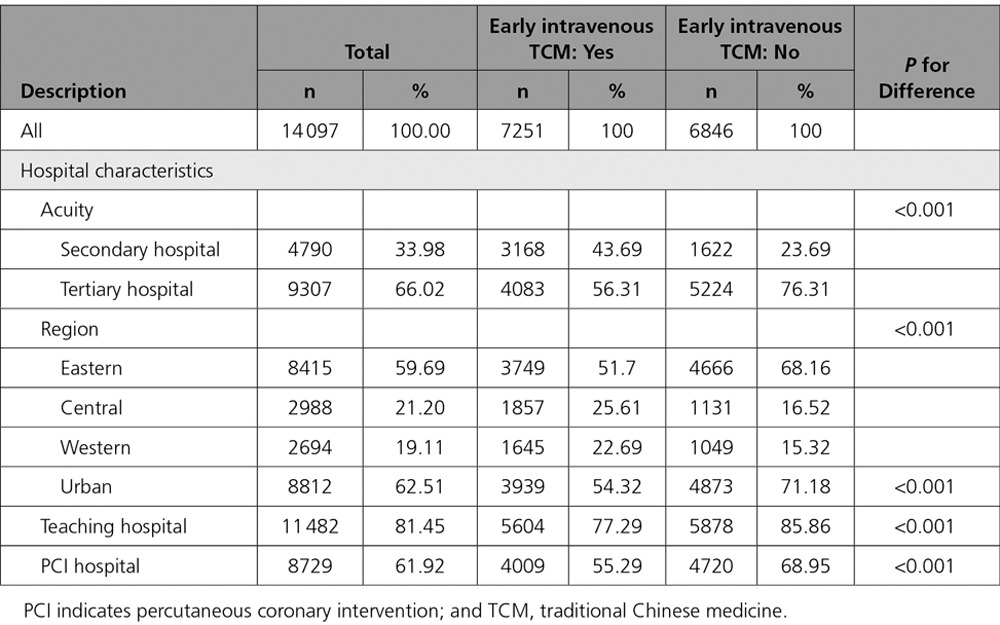
Hospital Characteristics Stratified by Use of intravenous TCM Within 24 Hours

In hierarchical logistic regression models, patient factors were not strongly associated with early intravenous TCM (Table III in the Data Supplement). However, patients treated in secondary hospitals were more likely to receive early intravenous TCM than patients treated in tertiary hospitals (OR: 2.85; 95% confidence interval: 1.98–4.11). Overall, we observed substantial variation in the use of early intravenous TCM across hospitals (median OR=2.84), suggesting that the average odds of a patient receiving early intravenous TCM at one random hospital versus another varied ≈3-fold after adjusting for patient and treatment characteristics (Table III in the Data Supplement). Fifty-five percent (55%) of the variance in use of early intravenous TCM among patients was explained by the nested effect (or clustering) of patients among hospitals (intra-class correlation: 0.55).

### Association of Early Intravenous TCM With In-Patient Outcomes

Patients receiving early intravenous TCM, compared with those who did not, had slightly lower rates of in-hospital bleeding (6.2% versus 7.4%, *P*<0.01; OR and 95% confidence interval: 0.82 [0.72, 0.94]) and similar rates of in-hospital mortality or treatment withdrawal (11.8% versus 11.0%, *P*=0.14; OR and 95% confidence interval: 1.08 [0.98, 1.20]). In the fully adjusted hierarchical models, there was no association with receipt of early intravenous TCM and in-patient bleeding or with mortality or treatment withdrawal (Table 5). Outcomes by type of TCM were examined both in unadjusted and multivariable hierarchical models, and there was no significant associations between use of Salvia miltiorrhiza, Folium gingko, Panax notoginseng, other early intravenous TCM or >1 early intravenous TCM with in-hospital bleeding, mortality, or treatment withdrawal (Tables intravenous and V in the Data Supplement).

**Table 5.**
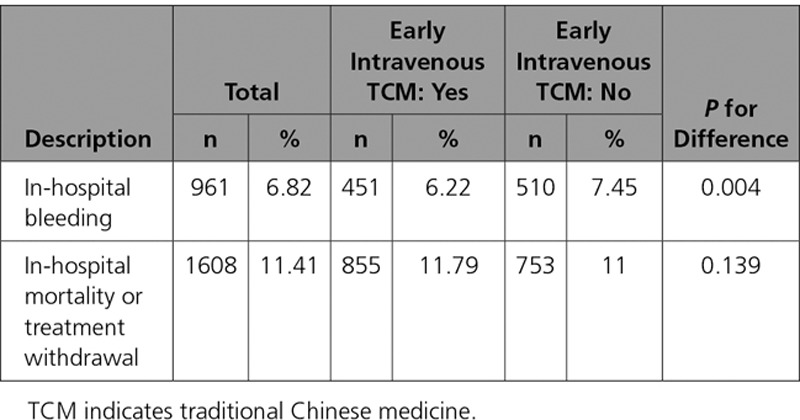
In-Hospital Events Stratified by Use of Intravenous TCM Within 24 Hours

## Discussion

In this nationally representative study, over half of patients hospitalized with AMI in modern Western medicine hospitals in China received intravenous TCM within the first 24 hours of hospitalization, a 20% absolute increase from 2001. Use of TCM was largely a function of hospital practice. Although early intravenous TCM was used in nearly all hospitals, secondary hospitals (often without PCI capability and located in rural regions) were more likely to use TCM. Still, about half of patients cared for in tertiary hospitals, and two thirds of patients cared for in teaching hospitals received early intravenous TCM. We did not observe differences in use of early intravenous TCM based on patient characteristics. We also did not find evidence that TCM was being used in lieu of evidence-based therapies; instead, our findings suggest that TCM is used as a complementary therapy in the early management of AMI. Use of TCM as a complementary therapy varied by hospital. Specifically, patients with statistically identical characteristics and clinical management, on average, were ≈3× more likely to receive early intravenous TCM at some hospitals versus others. Finally, in an exploratory analysis, we did not observe signals of harm or benefit with respect to in-hospital bleeding and in-patient mortality or treatment withdrawal. Our findings are consistent with larger national trends showing an increase in use of TCM for all diseases throughout China and reflect a complex culture in medicine that simultaneously encourages adherence to guidelines based on conventional treatment of AMI, including primary revascularization and medical therapy, along with integration of TCM.^11,19,20^

TCM has been an integral part of medicinal healing in China for centuries. In the 1950s, to preserve the tradition of TCM, the Chinese government invested in studies of TCM, and in 1958, they declared that TCM would be given support equal to that of Western medicine.^14^ However, with the emergence of evidence-based approaches for treating AMI in the 1980s and early 1990s, we expected to find a decline in the use of TCM, especially among Western hospitals in China which follow guidelines that are similar to the United States and Europe. In contrary, our data reveal that in a contemporary period, the use of TCM has increased. The rise in use may reflect even more government investment in preserving TCM, including more research on the effectiveness of TCM as well as broad insurance coverage for intravenous TCMs, which may influence the public’s perceptions about the benefits and safety of TCM.^14^ Several recent studies published in the Chinese literature and in complementary medicine journals show improved biological and clinical outcomes, including improved angiographic coronary blood flow and reflow,^21,22^ decreased stent restenosis, shorter hospital stays, less angina,^23^ fewer readmissions,^23^ improved quality of life, and improved short- and long-term mortality.^5,24^ In the case of Salvia miltiorrhiza/danshen, the most widely used TCM, there are reports of superior efficacy and fewer side effects than nitroglycerin for the treatment of angina.^25^ As such, Salvia miltiorrhiza/danshen may be more acceptable to and well tolerated by patients with angina. It is also one of 200+ TCMs included on the National Essential Drugs list, started in 2000 and last updated in 2017, which are made widely available at no or little cost. In fact, all of the TCMs that we observed to be in use were listed as class A or class B drugs, which are covered by insurance at 100% and 70% to 80% of cost, respectively. Nonetheless, at least 2 Cochrane reviews along with other systematic reviews have rendered the evidence either negative or inconclusive to support the use of TCM, including the use of Salvia miltiorrhiza/danshen for angina or coronary artery disease.^8,26,27^ Moreover, TCM is a small part of the curriculum of conventional Chinese medical schools, and use of Salvia miltiorrhiza/danshen to treat angina and other TCMs for the management of AMI are not recommended in Chinese clinical practice guidelines.^10^

Several observations suggest that hospital capacity may have played a role in the use of TCM. Hospitals without PCI capability and presumptively fewer resources were more likely to use TCM. Consistent with this, patients who received TCM were less likely to have undergone PCI although more likely to have received fibrinolysis. They were also less likely to have received an echocardiogram or a diagnostic cardiac catheterization. Local culture, at the hospital and of the region, may have also played a role. Secondary hospitals and hospitals located in the Central and Western regions of the country, which are typically poorer and tend to be less westernized, were more likely to use TCM.^28^

Importantly, we did not find evidence of TCM being used as a substitute for evidence-based therapies. For example, patients receiving early intravenous TCM were more likely to also receive aspirin and heparin in the first 24 hours and just as likely to receive clopidogrel and β-blocker therapy as compared with patients not receiving early intravenous TCM. Collectively, these findings suggest that TCM is not a last resort therapy; it does not seem to be used as a salvage therapy or as a replacement for conventional therapies, but rather, as a complementary therapy that is part of hospital culture in the treatment of AMI.

Several limitations should be considered in the interpretation of these findings. The classification of TCM assumed similar preparations and doses although there is likely substantial heterogeneity, and we did not capture prehospital usage. Nonetheless, the overwhelming majority of TCMs could be categorized into 1 of 5 main ingredients, with the majority of hospitals (87%) using Salvia miltiorrhiza/danshen in the first 24 hours of hospitalization, demonstrating a common approach to practice. Second, our approach to examining factors associated with TCM was based on data from a retrospective review of medical records, which may not capture the full spectrum of patient, physician, or hospital characteristics that might influence use of TCM. Third, our work examining associations of TCM with in-hospital outcomes is exploratory. Doses and formulations of intravenous TCM were not standardized, and we may not have had adequate power to exclude a clinically important association. In addition, the association of TCM with other short- and long-term outcomes, such as recurrent AMI, revascularization, and stroke, are important to assess. Ultimately, randomized trials would provide the most definitive evidence of the safety and efficacy of TCM. Fourth, our study is limited to China although the sample is representative of the care of a country with a fifth of the world’s population.

In conclusion, early intravenous TCM for the management of AMI occurs in modern Western medicine hospitals throughout China. Many types of TCM are used although the predominant formulation was with Salvia miltiorrhiza/danshen, touted for its properties as a vasodilator and anticoagulant. Although nearly all hospitals in our sample used early intravenous TCM, the proportion of patients treated varied by hospital but not by patient characteristics. Given the overall high use, there is an urgent need for the study of the safety and effectiveness of early intravenous TCM strategies in patients with AMI.

## Sources of Funding

At the time this study was conducted, Dr Spatz was supported by grant K12HS023000 from the Agency for Healthcare Research and Quality Patient-Centered Outcomes Research (PCOR) Institutional Mentored Career Development Program. This project was supported by the Research Special Fund for Public Welfare Industry of Health (201202025, 201502009) from the National Health and Family Planning Commission of China, the National Key Technology R&D Program (2013BAI09B01, 2015BAI12B01, 2015BAI12B02) from the Ministry of Science and Technology of China and the 111 Project (B16005). The funder had no role in study design, data collection, data analysis, data interpretation, or writing of the report.

## Disclosures

Drs Spatz and Krumholz report receiving support from the Centers for Medicare and Medicaid Services to develop and maintain performance measures that are used in public reporting programs. Dr Krumholz is a recipient of research agreements from Medtronic and from Johnson & Johnson (Janssen), through Yale, to develop methods of clinical trial data sharing; is the recipient of a grant from Medtronic, through Yale, to develop methods for postmarket surveillance of medical devices; chairs a cardiac scientific advisory board for UnitedHealth; is a participant/participant representative of the IBM Watson Health Life Sciences Board; is a member of the Advisory Board for Element Science and the Physician Advisory Board for Aetna; and is the founder of Hugo, a personal health information platform. Dr Masoudi has a contract with the American College of Cardiology as the Senior Medical Officer of the National Cardiovascular Data Registry. Dr Spertus has received grants from Gilead, Genentech, Lilly, Abbott Vascular, and Amorcyte; provides consulting services to Amgen, Novartis, Janssen, and Regeneron; owns the copyright to the Seattle Angina Questionnaire, Kansas City Cardiomyopathy Questionnaire, and Peripheral Artery Questionnaire; and has an equity interest in Health Outcomes Sciences. The other authors report no conflicts.

## Supplementary Material
