# Supplementary material for: Traditional Chinese Medicine for Acute Myocardial Infarction in Western Medicine Hospitals in China
Source: Circ Cardiovasc Qual Outcomes. 2018 Mar 16;11(3):e004190. doi: 10.1161/CIRCOUTCOMES.117.004190 (PMC5882246; doi:10.1161/CIRCOUTCOMES.117.004190)
Supplement: Supplementary file 1 [file hcq-11-e004190-s001.pdf]

SUPPLEMENTAL MATERIAL

Supplemental Table 1. Traditional Chinese Medicines and their potential cardiac effects

**Herbal Medicine**	**Cardiac Effect**		**Reference**
	Potential Benefit	Potential Harm	
Salvia miltiorrhiza<br><br>(*Other names:* Danshen; Hongshen; Renshen; Tanshinone; Red Ginseng; Ginseng)	Vasodilator; anticoagulant; may improve angina symptoms and survival after a heart attack	Constricts coronary arteries at high doses; may cause bleeding problems with warfarin, aspirin or other antiplatelet drugs; potentiates digoxin activity	Cheng 2007; Davidson et al. 2003; Stout et al. 2003; We et al. 2008; Bensky et al. 1987; Sang 1979
Follum gingko<br><br>(*Other names:* Ginkgo biloba)	May improve circulatory flow without appreciatively affecting blood pressure; decrease vascular resistance; decrease areas of ischemia; may help treat peripheral artery disease; potential anti-free radical action in myocardial ischemia-reperfusion injury; platelet inhibitor	Increased bleeding tendency; hemorrhagic stroke; may cause bleeding problems with warfarin, aspirin or other antiplatelet drugs	Davidson et al. 2003; Mashour et al. 1998; Stout et al. 2003; Kuller et al. 2010; Kudolo et al., 2003; Gardner et al. 2007
Panax notoginseng<br><br>(*Other names:* Pseudoginseng saponins, Xueshuantong)	May improve heart function in people with heart failure; possible small reduction in high blood pressure; treat angina and coronary artery disease; a calcium ion channel antagonist in vascular tissue; dilate coronary arteries	High blood pressure with overuse; platelet inhibitor; may cause bleeding problems with warfarin, aspirin or other antiplatelet drugs	Davidson et al. 2003; Stout et al. 2003; Mashour et al. 1998
Hirudin<br><br>(*Other names:* Lepirudin, Shuxuetong injection)	Acts as a direct thrombin inhibitor; may prevent myocardial infarction and refractory angina; may reduce thromboembolic complications in patients with heparin-induced thrombocytopenia	Reported effects include excess of major bleeding, particularly in acute coronary syndromes without ST elevation; may cause induction of anti-hirudin antibodies	Greinacher et al. 2001; OASIS-2 investigators 1999; Lubenow et al. 2005; Neuhaus et al. 1999
Erigeron Breviscapus	May act as a protein kinase C inhibitor; appears to be tolerated in healthy volunteers; limited information available	Limited information available	Ju et al 2015; Zhou et al. 2002

(*Other names:*<br>Erigeron breviscapus injection, Breviscapinun)			
Xuezhikang<br><br>(*Other lipid-lowering agents include*:, Taizhian, Hongquqingchunsu)	Improve lipoprotein regulation; protect endothelial function through anti-inflammatory activity; may decrease myocardial infarction and death from coronary heart disease	May increase serum creatinine levels beyond normal	Lu et al. 2008; Zhao et al. 2004; Du et al. 2006; Ye et al. 2007; Zhao et al. 2007; Lu et al. 2005; Liu et al. 2003

Supplemental Table 2. Comparison of TCM type, route, and timing of administration across three time periods

Description	Total		2001		2006		2011		P
	#	%	#	%	#	%	#	%	
All	14097	100.00	2101	100.00	4026	100.00	7970	100.00	
Any TCM									
In hospital	9424	66.85	1222	58.16	2608	64.78	5594	70.19	<0.001
IV In hospital	8664	61.46	1060	50.45	2398	59.56	5206	65.32	<0.001
Within 24 hours	7964	56.49	945	44.98	2172	53.95	4847	60.82	<0.001
IV within 24 hours	7251	51.44	802	38.17	1976	49.08	4473	56.12	<0.001
Salvia miltiorrhiza (danshen)									
In hospital	7103	50.39	939	44.69	1933	48.01	4231	53.09	<0.001
IV	6390	45.33	831	39.55	1712	42.52	3847	48.27	<0.001
Within 24 hours	5605	39.76	690	32.84	1493	37.08	3422	42.94	<0.001
IV within 24 hours	5004	35.50	603	28.70	1298	32.24	3103	38.93	<0.001
Folium ginkgo									
In hospital	1772	12.57	114	5.43	543	13.49	1115	13.99	<0.001
IV	1399	9.92	7	0.33	456	11.33	936	11.74	<0.001
Within 24 hours	1329	9.43	78	3.71	397	9.86	854	10.72	<0.001
IV within 24 hours	1063	7.54	6	0.29	333	8.27	724	9.08	<0.001
Panax notoginseng									
In hospital	2450	17.38	238	11.33	555	13.79	1657	20.79	<0.001
IV	2116	15.01	164	7.81	480	11.92	1472	18.47	<0.001
Within 24 hours	1930	13.69	177	8.42	422	10.48	1331	16.70	<0.001
IV within 24 hours	1717	12.18	129	6.14	379	9.41	1209	15.17	<0.001
Breviscapinun									
In hospital	515	3.65	92	4.38	170	4.22	253	3.17	0.0011
IV	474	3.36	90	4.28	161	4.00	223	2.80	<0.001
Within 24 hours	346	2.45	67	3.19	118	2.93	161	2.02	0.0002
IV within 24 hours	312	2.21	66	3.14	111	2.76	135	1.69	<0.001
Xuezhikang									
In hospital	105	0.74	44	2.09	41	1.02	20	0.25	<0.001
IV									
Within 24 hours	72	0.51	33	1.57	28	0.70	11	0.14	<0.001

IV within 24 hours									
Puerarin									
In hospital	552	3.92	119	5.66	248	6.16	185	2.32	<0.001
IV	526	3.73	112	5.33	242	6.01	172	2.16	<0.001
Within 24 hours	390	2.77	72	3.43	185	4.60	133	1.67	<0.001
IV within 24 hours	374	2.65	68	3.24	181	4.50	125	1.57	<0.001
Kyushin Pills									
In hospital	296	2.10	67	3.19	81	2.01	148	1.86	0.0007
IV	4	0.03	1	0.05	2	0.05	1	0.01	0.2576
Within 24 hours	146	1.04	35	1.67	42	1.04	69	0.87	0.0025
IV within 24 hours									

Supplemental Table 3. Factors Associated with Use of IV TCM within 24 Hours

Label	Estimate	StdErr	OR	LOR	UOR
Intercept	-0.35	0.22			
Year (ref: 2011)					
2001	-0.67	0.07	0.51	0.44	0.59
2006	-0.31	0.05	0.74	0.67	0.81
Clinical Presentation					
Presented within 3 hours of symptom onset	0.10	0.06	1.10	0.97	1.25
Presented within 3 to 15 hours of symptom onset	0.14	0.05	1.15	1.04	1.28
Presented within 15 to 72 hours of symptom onset	-0.01	0.06	0.99	0.88	1.11
Heart Failure on Presentation	-0.09	0.04	0.91	0.84	1.00
Pneumonia on Presentation	-0.15	0.07	0.86	0.75	0.99
Acute Stroke on Presentation	0.66	0.18	1.93	1.35	2.75
HR<60	0.00	0.00	1.00	0.99	1.00
HR>90	0.00	0.00	1.00	0.99	1.00
SBP<100	0.47	0.07	1.60	1.40	1.82
SBP>160	-0.19	0.07	0.83	0.73	0.95
Past Medical History					
Hypertension	-0.12	0.04	0.89	0.82	0.97
Stroke	0.15	0.06	1.16	1.02	1.31
Prior Aspirin	-0.19	0.09	0.82	0.69	0.99
Clinical Management (re: not performed/received)					
Echocardiography	0.20	0.06	1.22	1.08	1.38
Primary PCI	-0.26	0.08	0.77	0.66	0.90
Fibrinolytic therapy	0.11	0.06	1.12	1.00	1.25
Aspirin	0.43	0.07	1.54	1.35	1.75
Heparin	0.46	0.05	1.58	1.43	1.75
Clopidogrel	0.24	0.06	1.27	1.13	1.43
Hospital Characteristics					
Secondary hospital	1.05	0.19	2.85	1.97	4.11
Between Hospital Variance	1.20	0.15			
Median Odds Ratio	2.84			2.46	3.21

*Intraclass Coefficient (ICC): 0.547

Supplemental Table 4. Independent Association of Use of IV TCM within 24 Hours with In-patient Outcomes

Description	Bleeding				Death				Withdrawal of treatment			
	OR	LOR	UOR	P	OR	LOR	UOR	P	OR	LOR	UOR	P
Any TCM	1.09	0.94	1.27	0.245	1.04	0.90	1.20	0.585	1.08	0.88	1.33	0.445
Salvia Miltiorrhiza	1.19	1.00	1.41	0.045	1.16	0.99	1.35	0.070	1.05	0.83	1.32	0.687
Folium Gingko	0.96	0.67	1.37	0.804	0.73	0.51	1.06	0.095	0.65	0.36	1.16	0.143
Panax notoginseng	0.90	0.67	1.22	0.514	0.86	0.65	1.14	0.299	1.25	0.86	1.80	0.242
Other TCM	0.91	0.60	1.39	0.668	0.96	0.66	1.40	0.825	1.18	0.70	1.98	0.534
>1 TCM†	1.06	0.79	1.42	0.705	0.96	0.74	1.25	0.778	1.32	0.96	1.82	0.090

*Compared with No IV TCM within the first 24 hours of hospitalization

†More than one group of the specified four groups above.

Supplemental Table 5. Independent Association of Type of IV TCM within 24 Hours with In-patient Outcomes*

Description	Salvia miltiorrhiza (danshen)				Folium ginkgo				Panax notoginseng				Breviscapinun				Puerarin			
	**OR**	**LOR**	**UOR**	**P**	**OR**	**LOR**	**UOR**	**P**	**OR**	**LOR**	**UOR**	**P**	**OR**	**LOR**	**UOR**	**P**	**OR**	**LOR**	**UOR**	**P**
**Bleeding**																				
Unadjusted	1.17	0.99	1.37	0.07	0.97	0.68	1.38	0.85	0.88	0.65	1.18	0.39	0.82	0.54	1.24	0.35	1.05	0.79	1.39	0.75
Adjusted: #1	1.14	0.97	1.35	0.11	0.91	0.63	1.30	0.59	0.84	0.62	1.14	0.26	0.84	0.55	1.28	0.42	0.99	0.74	1.32	0.95
Adjusted: #2	1.11	0.94	1.31	0.20	0.90	0.63	1.29	0.58	0.84	0.62	1.14	0.26	0.86	0.56	1.30	0.47	0.95	0.71	1.26	0.71
Adjusted: #3	1.12	0.95	1.32	0.17	0.90	0.63	1.29	0.56	0.85	0.63	1.15	0.29	0.86	0.56	1.30	0.47	0.96	0.72	1.28	0.79
Adjusted: #4	1.12	0.95	1.32	0.18	0.91	0.64	1.31	0.62	0.85	0.63	1.14	0.27	0.86	0.57	1.31	0.49	0.96	0.72	1.28	0.77
Adjusted: #5	1.15	0.98	1.36	0.10	0.93	0.65	1.34	0.70	0.88	0.65	1.19	0.41	0.88	0.58	1.34	0.56	1.00	0.75	1.33	0.98
Adjusted: #6	1.19	1.00	1.41	0.04	0.96	0.67	1.37	0.80	0.90	0.67	1.22	0.51	0.91	0.60	1.39	0.67	1.06	0.79	1.42	0.70
**Death**																				
Unadjusted	1.12	0.97	1.30	0.12	0.63	0.45	0.89	0.01	0.73	0.55	0.95	0.02	0.95	0.66	1.36	0.78	0.91	0.72	1.16	0.46
Adjusted: #1	1.14	0.99	1.32	0.07	0.67	0.47	0.96	0.03	0.73	0.56	0.96	0.03	0.89	0.62	1.28	0.53	0.91	0.71	1.16	0.45
Adjusted: #2	1.08	0.93	1.25	0.34	0.71	0.50	1.02	0.06	0.78	0.59	1.03	0.08	0.92	0.63	1.33	0.65	0.89	0.69	1.15	0.37
Adjusted: #3	1.08	0.93	1.26	0.30	0.71	0.50	1.01	0.06	0.77	0.58	1.02	0.07	0.91	0.63	1.33	0.62	0.90	0.70	1.16	0.41
Adjusted: #4	1.03	0.88	1.21	0.67	0.65	0.45	0.94	0.02	0.75	0.56	1.00	0.05	0.85	0.58	1.24	0.40	0.80	0.61	1.03	0.09
Adjusted: #5	1.12	0.96	1.31	0.16	0.71	0.49	1.02	0.07	0.84	0.63	1.12	0.23	0.93	0.64	1.35	0.69	0.91	0.70	1.17	0.45
Adjusted: #6	1.16	0.99	1.35	0.07	0.73	0.51	1.06	0.10	0.86	0.65	1.14	0.30	0.96	0.66	1.40	0.82	0.96	0.74	1.25	0.78
**Withdrawal of treatment**																				
Unadjusted	1.05	0.84	1.30	0.68	0.61	0.35	1.06	0.08	1.03	0.72	1.46	0.88	1.05	0.64	1.71	0.85	1.38	1.02	1.86	0.03
Adjusted: #1	1.05	0.84	1.31	0.66	0.61	0.35	1.07	0.08	1.00	0.70	1.43	0.99	1.08	0.66	1.78	0.75	1.31	0.97	1.78	0.08
Adjusted: #2	1.01	0.81	1.27	0.91	0.63	0.36	1.12	0.11	1.14	0.79	1.64	0.48	1.10	0.66	1.83	0.71	1.30	0.95	1.77	0.10
Adjusted: #3	1.01	0.81	1.26	0.92	0.62	0.35	1.10	0.11	1.16	0.81	1.67	0.42	1.12	0.68	1.86	0.66	1.33	0.98	1.82	0.07
Adjusted: #4	0.99	0.79	1.24	0.94	0.60	0.34	1.08	0.09	1.12	0.78	1.61	0.54	1.10	0.66	1.83	0.71	1.20	0.88	1.65	0.25
Adjusted: #5	1.07	0.85	1.34	0.58	0.65	0.37	1.17	0.15	1.26	0.87	1.82	0.22	1.17	0.70	1.96	0.55	1.34	0.97	1.85	0.07
Adjusted: #6	1.05	0.83	1.32	0.69	0.65	0.36	1.16	0.14	1.25	0.86	1.80	0.24	1.18	0.70	1.98	0.53	1.32	0.96	1.82	0.09

#1 -- Demographics (Year, Age Group)
#2 -- #1 and admission characteristics (STEMI and MiniGRACE)
#3 -- #2 and medical history (Angina or Coronary Heart Disease, AMI, Smoking, HTN, DM, and Dyslipidemia).
#4 -- #3 and diagnosis tests or procedures (Echocardiography, LVEF, use of TN, PPCI, and LYSIS)
#5 -- #4 and Medications (Aspirin, Heparin, Clopidogrel).
#6 -- #5 and hospital characteristics (Acuity, PCI capability)

*Compared with No IV TCM within the first 24 hours of hospitalization
